# Histogram of Apparent Diffusion Coefficient to Evaluate the Activity of Thyroid‐Associated Ophthalmopathy

**DOI:** 10.1002/iid3.70131

**Published:** 2025-01-21

**Authors:** Defu Li, Tingting Zhu, Yujin Wang

**Affiliations:** ^1^ Department of Radiology Fuyong People's Hospital of Baoan District Shenzhen China; ^2^ Department of Radiology, Tongji Hospital, Tongji Medical College Huazhong University of Science and Technology Wuhan China

**Keywords:** apparent diffusion coefficient, clinical activity score, extraocular muscle, intravoxel incoherent motion, thyroid‐associated ophthalmopathy, turbo spin echo

## Abstract

**Objective:**

This study aimed to evaluate the activity of extraocular muscles (EOMs) in patients with thyroid‐associated ophthalmopathy (TAO) using turbo spin echo imaging. By analyzing tissue heterogeneity, apparent diffusion coefficient (ADC) histogram analysis offers enhanced insights into edema within the EOMs.

**Methods:**

Eighty‐eight patients with TAO were retrospectively evaluated and allocated into active (*n* = 24, clinical activity score [CAS] ≥ 3) and inactive (*n* = 64, CAS < 3) groups. The parameter values of the ADC histogram of EOMs were measured; the efficacy of ADC histograms in distinguishing between TAO activity and inactivity was assessed using receiver operating characteristic curves. Multifactorial logistic regression was used to determine active TAO predictors.

**Results:**

The minimum, maximum, median, mean; and 1st, 5th, 10th, 25th, 75th, 90th, 95th, and 99th percentiles of the ADC histograms were higher in patients with active than that in participants with inactive TAO. The area under the curve (AUC) of the 10th percentile of the ADC histogram and the median distinguishing between active and inactive TAOs were both 0.791 (both *p* < 0.05), and the AUCs of the combined model of age, sex, smoking, and the 10th percentile in the ADC histogram were better than those of their individual models and the combined model of age, sex, and smoking (all *p* < 0.05). Smoking and male sex, along with the median > 1.26 μm^2^/s, entropy > 4.03, and standard deviation (SD) > 0.4 of the ADC histogram, were significant predictors of TAO activity, with odds ratios of 2.741 and 6.806, 5.070, 2.652, and 2.197, respectively (all *p* < 0.05).

**Conclusion:**

ADC histograms provide a new method for distinguishing active from inactive TAO, and the 10th percentile enhances the clinical diagnosis of active TAO. In addition to male sex and smoking, an ADC histogram median > 1.26 μm²/s, entropy > 4.03, or SD > 0.4 may also predict active TAO.

## Graphical abstract

We assessed extraocular muscles activity in thyroid‐associated ophthalmopathy cases. Apparent diffusion coefficient histograms were useful tools. Particularly, they were useful for clinically understanding inflammation.

## 1 Introduction

Thyroid‐associated ophthalmopathy (TAO), also known as thyroid eye disease or Graves' ophthalmopathy, is a chronic autoimmune inflammatory disease caused by autoantibodies against the thyrotropin receptor and is often associated with Graves' disease [1]. Over 90% of patients with TAO have Graves' disease [1]. Moreover, TAO occurs in approximately 40% of patients with Graves' disease [2]. Typical symptoms of TAO include proptosis, orbital pain, extraocular muscle (EOM) dysfunction, diplopia, and even blindness [3].

According to disease progression, TAO can be divided into active and inactive phases. The active phase of TAO is characterized by intraorbital inflammatory cell infiltration, activation of fibroblasts, and massive production of glycosaminoglycans, leading to interstitial tissue edema and swelling of EOMs. The inactive phase is characterized by interstitial fibrosis, fat infiltration, and collagen deposition [4]. The accurate determination of TAO inflammatory activity directly affects the choice of clinical treatment options and prognosis; assessment of the TAO active phase is mainly based on the clinical activity score (CAS) (Active: CAS ≥ 3; inactive: CAS < 3) [5]. The CAS is a well‐established clinical scoring system for assessing TAO activity. However, it is highly subjective, and its results are highly dependent on the examiner [6].

Quantitative magnetic resonance imaging (MRI) techniques are increasingly used in diagnosing TAO, providing detailed insights into morphological and functional changes in the orbit [7]. MRI holds significant potential for unraveling the complex biology of TAO [8]. Diffusion‐weighted imaging (DWI) has been applied to assess TAO activity [9, 10]. However, conventional apparent diffusion coefficients (ADCs) analysis primarily focuses on the mean values, which fail to adequately capture tissue heterogeneity.

Volume histogram analysis, based on first‐order statistical texture analysis, evaluates tissue heterogeneity by analyzing the gray‐scale voxel distribution in the volume of interest [11]. ADC histogram multidata analysis extends this by offering additional data on tissue heterogeneity, thereby providing a more comprehensive assessment of EOM edema. Therefore, in this study, we aimed to calculate ADC based on DWI theory to determine the parameter differences between patients with active and inactive TAO.

## 2 Materials and Methods

### 2.1 Population

The Ethics Committee of Fuyong People's Hospital of Baoan District, Shenzhen, China, approved this retrospective study (approval number: KY‐2022‐10‐0) and waived the need for informed consent. Ninety‐five consecutive patients with TAO with detailed magnetic resonance (MR) data from our hospital between June 2020 and October 2023 were retrospectively included. The inclusion criteria included patients meeting the Bartley diagnostic criteria for TAO [12], aged > 18 years, undergoing DWI, and having no prior ocular therapies before MRI, such as hormones or immunosuppression. Exclusion criteria were the presence of comorbid ocular diseases or severe image artifacts that rendered diagnosis impossible.

To minimize data bias, several measures were implemented. A double‐blind design ensured that neither the participants nor the implementers were aware of subgroup classifications, reducing subjective influence. Strict inclusion and exclusion criteria were applied to ensure the study population was representative of the target population characteristics. Continuous inclusion was employed to avoid selection bias from artificial participant selection. Data collection methods were harmonized to ensure consistency across all patient groups. Finally, study variables and ADC histogram data were clearly defined and measured repeatedly, with checks to confirm data accuracy and reliability.

### 2.2 MR Scanning Sequence and Parameters

All participants underwent scanning using a 1.5 T MRI scanner (Prodiva, Philips Healthcare, Bets, the Netherlands) equipped with a 16‐channel phased‐array head‐and‐neck coil. The participants were placed in the supine position on the scanning bed with their eyes closed naturally. The MR sequences for orbital imaging included axial and coronal T2‐weighted imaging (WI), axial T1WI, and coronal DWI (Table 1). The coronal scanning baseline was perpendicular to the sagittal plane of the body, and the scanning areas were the anterior rim, including the lens, and the posterior rim, including the orbital apex.

**Table 1:** Detailed MR sequence parameters.

	Axial T1WI	Axial T2WI	Coronal T2WI	Coronal DWI
Fast imaging mode	TFE	TSE	TSE	TSE
Repetition time (ms)	599	3841	3000	1947
Echo time (ms)	14	120	100	73
Slice thickness (mm)	3	3	3	4
FS	mDixon	mDixon	mDixon	SPAIR
Flip angle	90°	90°	90°	90°
Number of excitations	1.5	1	1	1, 2, 3, 4, 4
Spacing (ms)	0.3	0.3	0.3	0.4
Pixel size (mm)	0.8 × 0.9	0.85 × 0.96	0.65 × 0.77	2.2 × 2.54
Field of view (mm)	160 × 160	160 × 160	160 × 160	160 × 160
Bandwidth (Hz)	664.9	229.7	258.1	268.3
Acquisition matrix	200 × 177	188 × 163	276×252	72 × 64
Acquisition duration (s)	2 min and 9 s	1 min and 44 s	2 min and 30 s	10 min and 23 s
*b* values (sec/mm^2^)				0, 15, 90, 150, 800

Abbreviations: FS, fat‐suppressed; mDixon, modified Dixon; MR, magnetic resonance; SPAIR, SPectral Attenuated Inversion Recovery; TFE, turbo field echo; TSE, turbo spin echo; WI, weighted imaging.

### 2.3 Image Analysis

**Figure 1:**
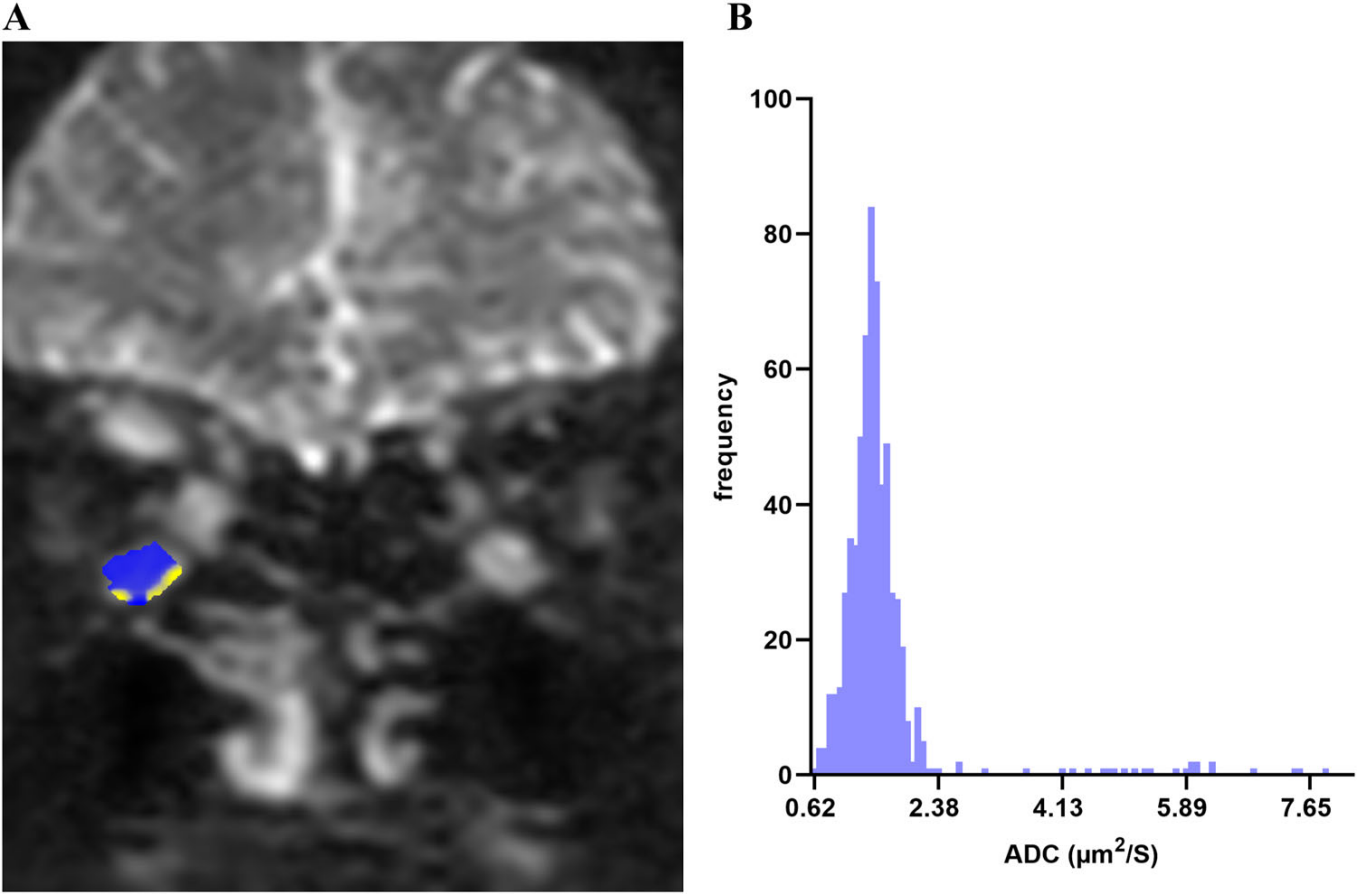
Patient with active TAO (male, 46 years). A. DWI coronal image with *b* = 0, showing bilateral inflammatory edema of the medial and inferior rectus muscles and the right superior rectus muscle, with markedly increased signal. The blue‐yellow area of the right inferior rectus muscle is the area of interest at this level. (B) Histogram of the ADC of the right inferior rectus muscle, reflecting the distribution of each ADC value. ADC, apparent diffusion coefficient; DWI, diffusion‐weighted imaging; TAO, thyroid‐associated ophthalmopathy.

ADC histograms of turbo spin echo (TSE)‐DWI were extracted using the FireVoxel software (https://firevoxel.org/). The histograms include the minimum, 1st, 5th, 10th, 25th, 75th, 90th, 95th, 99th, and maximum percentiles; median; mean; standard deviation (SD); skewness; kurtosis; and entropy. Two radiologists (D.L. and T.Z.) with > 5 years of experience in diagnostic orbital MR, blinded to the patient's clinical data, measured the ADC histograms of eight EOMs of the external, inferior, internal, and superior rectus muscles in both eyes of the patient. The region of interest was manually outlined along the edges of the rectus externus, rectus inferior, rectus internus, and rectus supraspinatus on each coronal image from front to back, with a b‐value of 0. The rectus inferior and rectus internus were protected from inflammation or gas in the maxillary sinus or sieve sinus; the rectus supraspinatus was protected from cerebrospinal fluid, as shown in Figure 1. After an interval of 1 month, both physicians repeated the measurements to assess intra‐ and interobserver agreements.

### 2.4 Statistical Analysis

All data were statistically analyzed using SPSS 25.0. The Kolmogorov–Smirnov test was used to analyze the conformity of the measures to normal distribution. Measurement data conforming to normal distribution are expressed as mean ± SD, and count data are expressed as the number of cases (*n*, %). The area under the curve (AUC), sensitivity, and specificity of the EOM ADC histogram for distinguishing TAO activity from inactivity were analyzed using the receiver operating characteristic (ROC) curve, and the cutoff value was determined based on the maximum Youden index. The Delong test was used to compare between differences in AUC using the Medcalc software (version 20.0, Ostend, Belgium).

The potential predictors of active TAO were determined using multifactor logistic regression. First, linear regression was used to assess collinearity among variables, with multicollinearity determined based on variance_inflation_factor (VIF) values. A VIF greater than 10 indicated strong collinearity between a variable and others, leading to the exclusion of that variable. Next, using cutoff values derived from ROC curve analysis, each variable was dichotomized. Independent variables were then analyzed using one‐way binary logistic regression to identify those significantly associated with the dependent variables at *p* < 0.1. Finally, variables with *p* < 0.1, along with clinically important variables, were included in a multivariate binary logistic regression model, analyzed using the enter method. Correlations between ADC histograms and CASs were analyzed using Spearman's test to determine the strength of correlations based on correlation coefficients (strong, *r* ≥ 0.7; moderate, 0.5 ≤ *r* < 0.7; and summed *r* < 0.5 difference). Inter‐ and intraobserver agreements were analyzed using the intragroup correlation coefficient (> 0.80 was considered good agreement). *p* < 0.05 was considered statistically significant.

## 3 Results

### 3.1 Patient Clinical Baseline

Seven patients were excluded (two with a history of ocular trauma, two with ocular tumors, and three with motion artifacts in the MR images that could not be observed). Finally, 88 patients with TAO (176 eyes in total; 45 male and 43 female patients; mean age: 35.4 ± 10.1 years) were included in this study. Patients with active TAO were less likely to be female, were older, and had a higher prevalence of smoking history compared to those with inactive TAO (all *p* < 0.001) (Table 2). Based on the CAS criteria [5], patients with TAO were classified into two groups: the active group (CAS ≥ 3 points; 24 cases, 48 eyes, 192 EOMs) and the inactive group (CAS < 3 points; 64 cases, 128 eyes, 512 EOMs).

**Table 2:** Clinical characteristics of patients with thyroid‐associated ophthalmopathy.

	Inactive	Active	*p*
Female (%)	40 (93)	3 (12.5)	< 0.001
Age (years)	32.7 ± 8.3	42.7 ± 11.0	< 0.001
Course of disease (months)	3.5 (1.75, 7.75)	3 (1.5, 8)	0.337
Smoking history (%)	15 (23.4)	18 (75)	< 0.001
Thyrotropin (uIU/mL)	0.005 (0.005, 1.096)	1.71 (0.1295, 5.91)	0.838
Thyroid‐stimulating hormone receptor antibody (IU/L)	9.05 (3.3, 14.05)	8.7 (2.65, 17.8)	0.659
Thyroid peroxidase antibody (IU/mL)	46.4 (9, 600)	9 (9, 27.25)	0.187
Thyroglobulin antibodies (IU/mL)	18.75 (14.33, 546.25)	18.8 (15.45, 23.1)	0.500
Triiodothyronine (pg/mL)	2.1 (1.39, 3.97)	1.06 (1.01, 1.44)	0.068
Tetraiodothyronine (pg/mL)	12.55 (8.28, 14.28)	6.39 (5.6, 9.02)	0.062
Course of disease (months)	3.5 (1.75, 7.75)	3 (1.5, 8)	0.337

### 3.2 ADC Histogram Characteristics of EOMs

The minimum, 1st, 5th, 15th, 25th, 75th, 90th, 95th, 99th, and maximum percentiles; median; and mean values of the ADC histograms were significantly greater in the active than that in the inactive group (all, *p* < 0.05), whereas skewness, kurtosis, and entropy were not significantly different between the two groups (Table 3).

**Table 3:** Comparison of ADC histograms of extraocular muscles in patients with active and inactive thyroid‐associated orbitopathy.

	Inactive	Active	*p*
Median (μm^2^/s)	0.97 (0.82, 1.13)	1.31 (1.04, 1.53)	< 0.001
Minimum (μm^2^/s)	0.21 (0, 0.41)	0.45 (0.21, 0.71)	< 0.001
Maximum (μm^2^/s)	2.22 (1.68, 4.41)	3.2 (2.12, 5.4)	< 0.001
Mean (μm^2^/s)	1.03 (0.85, 1.21)	1.4 (1.09, 1.66)	< 0.001
Standard deviation	0.36 (0.28, 0.55)	0.41 (0.3, 0.65)	0.023
Skewness	0.69 (0.15, 2.0)	0.85 (0.16, 2.14)	0.410
Kurtosis	1.07 (−0.018, 5.59)	1.3 (0.10, 6.94)	0.204
Entropy	3.86 (3.52, 4.01)	3.89 (3.55, 4.05)	0.209
Percentile			
1st (μm^2^/s)	0.32 (0.13, 0.51)	0.63 (0.35, 0.87)	< 0.001
5th (μm^2^/s)	0.49 (0.32, 0.67)	0.79 (0.55, 1.06)	< 0.001
10th (μm^2^/s)	0.6 (0.44, 0.76)	0.9 (0.66, 1.19)	< 0.001
25th (μm^2^/s)	0.78 (0.61, 0.95)	1.08 (0.82, 1.34)	< 0.001
75th (μm^2^/s)	1.2 (1.04, 1.39)	1.55 (1.25, 1.80)	< 0.001
90th (μm^2^/s)	1.45 (1.25, 1.71)	1.85 (1.5, 2.15)	< 0.001
95th (μm^2^/s)	1.66 (1.39, 2.07)	2.01 (1.66, 2.63)	< 0.001
99th (μm^2^/s)	2.1 (1.63, 3.9)	2.57 (2.02, 4.61)	< 0.001

*Note:* 1st, 5th, 10th, 25th, 75th, 90th, 95th, and 99th: 1st, 5th, 10th, 25th, 75th, 90th, 95th, and 99th percentiles of ADC histograms.

Abbreviation: ADC, apparent diffusion coefficient.

### 3.3 Diagnostic Efficacy Analysis of ADC Histogram to Distinguish TAO Activity From Inactivity

**Figure 2:**
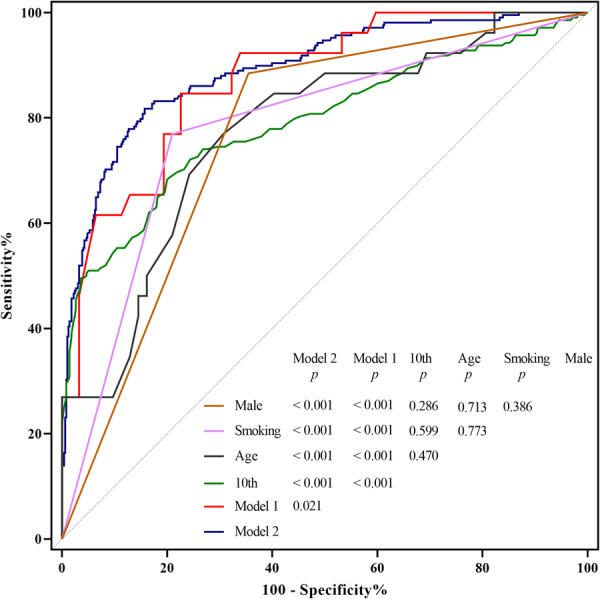
Comparison of area under the curves between models for differentiating between active and inactive thyroid‐related ophthalmopathy. 10th, 10th percentile of apparent diffusion coefficient histograms; *p*, *p* value.

Age, smoking history, and male sex, along with the 10th percentile, median, and mean of ADC histograms, differentiated active from inactive TAO, with AUC values of 0.774, 0.780, and 0.765, 0.791, 0.791, and 0.765, respectively. The optimal ADC cutoff values were > 0.78, > 1.26, > 1.27, and > 1.31 μm^2^/s, respectively (all *p* < 0.05; Table 4). Model 1, which incorporates age, sex, and smoking history, demonstrated a significantly higher AUC compared to models that considered only age, sex, smoking, or the 10th percentile in the ADC histogram individually (all *p* < 0.05). Furthermore, Model 2, which adds the 10th percentile in the ADC histogram to Model 1, achieved a significantly better AUC than Model 1 and all individual models (all *p* < 0.05; Figure 2).

**Table 4:** Diagnostic efficacy analysis of ADC histograms and clinical characteristics to differentiate between active and inactive TAO.

	AUC	95% CI	Cutoff value	Sensitivity	Specificity	*p*
Age	0.774	0.741–0.804	> 35 years	76.92	69.35	< 0.001
Smoking history	0.780	0.717–0.799		76.92	79.03	< 0.001
Male	0.765	0.732–0.796		88.46	64.52	< 0.001
ADC						
Median	0.791	0.759–0.820	> 1.26 μm^2^/s	57.21	91.53	< 0.001
Minimum	0.722	0.687–0.755	> 0.58 μm^2^/s	42.31	92.54	< 0.001
Maximum	0.653	0.616–0.688	> 1.99 μm^2^/s	83.17	44.35	< 0.001
Mean	0.765	0.731–0.795	> 1.31 μm^2^/s	60.10	84.88	< 0.001
Percentile						
1st	0.759	0.726–0.790	> 0.55 μm^2^/s	61.06	83.47	< 0.001
5th	0.784	0.752–0.814	> 0.65 μm^2^/s	70.67	76.21	< 0.001
10th	0.791	0.759–0.821	> 0.78 μm^2^/s	68.27	80.04	< 0.001
25th	0.788	0.756–0.818	> 1.09 μm^2^/s	53.37	93.15	< 0.001
75th	0.781	0.748–0.811	> 1.47 μm^2^/s	61.06	85.89	< 0.001
90th	0.739	0.705–0.771	> 1.65 μm^2^/s	67.79	73.59	< 0.001
95th	0.697	0.661–0.730	> 1.88 μm^2^/s	64.42	70.36	< 0.001
99th	0.640	0.603–0.675	> 1.94 μm^2^/s	81.73	45.16	< 0.001
Model 1	0.874	0.847–0.897		84.62	77.42	< 0.001
Model 2	0.892	0.867–0.914		81.73	84.27	< 0.001

*Note:* 1st, 5th, 10th, 25th, 75th, 90th, 95th and 99th: 1st, 5th, 10th, 25th, 75th, 90th, 95th, and 99th percentiles of ADC histograms.

Abbreviations: ADC, apparent diffusion coefficient; AUC, area under curve; CI, confidence interval; Model 1, joint model of age, masculinity, and smoking; Model 2, Model 1 + 10th.

### 3.4 Analysis of Predictors of Active TAO

Multifactorial logistic regression analysis showed that smoking and male sex, along with the median, entropy, and SD of the ADC histogram, were significant predicted of TAO activity, with odds ratios of 2.741 and 6.806, 5.070, 2.652, and 2.197, respectively (all *p* < 0.05; Table 5).

**Table 5:** Multifactorial logistic regression analysis of ADC histograms and clinical characteristics to predict activity in patients with TAO.

	*β*	SE	Wals	OR	95% CI	*p*
Age > 35 years	0.182	0.232	0.613	1.199	0.761–1.891	0.434
Smoking history	1.008	0.275	13.473	2.741	1.600–4.697	< 0.001
Male	1.918	0.321	35.726	6.806	3.629–12.765	< 0.001
ADC						
Entropy > 4.03	0.975	0.293	11.108	2.652	1.494–4.706	0.001
Standard deviation > 0.4	0.787	0.311	6.382	2.197	1.193–4.0445	0.012
Median > 1.26 μm^2^/s	1.623	0.525	9.574	5.070	1.813–14.175	0.002
Mean > 1.25 μm^2^/s	−0.550	0.549	1.006	0.577	0.197–1.691	0.316
5th > 0.65 μm^2^/s	0.208	0.553	0.142	1.232	0.417–3.642	0.706
10th > 0.78 μm^2^/s	0.733	0.621	1.393	2.080	0.6164–7.022	0.238
25th > 0.89 μm^2^/s	0.172	0.465	0.137	1.188	0.477–2.956	0.711
25th > 1.47 μm^2^/s	0.435	0.590	0.544	1.545	0.486–4.905	0.461
90th > 1.64 μm^2^/s	−0.132	0.464	0.081	0.876	0.353–2.1767	0.776
95th > 1.77 μm^2^/s	0.024	0.408	0.004	1.025	0.461–2.279	0.953

*Note:* 1st, 5th, 10th, 25th, 75th, 90th, 95th and 99th: 1st, 5th, 10th, 25th, 75th, 90th, 95th, and 99th percentiles of ADC histograms.

Abbreviations: ADC, apparent diffusion coefficient; *β*, regression coefficient; CI, confidence interval; OR, odds ratio; SE, standard error.

### 3.5 Correlation Between ADC Histograms and CASs of EOMs in Patients With TAO

Spearman's correlation analysis showed that the SD, kurtosis, entropy, and 1st percentile of the ADC histogram did not correlate with the CAS (all *p* > 0.05); the other ADC values correlated poorly with the CAS (*r* ranged from −0.214 to 0.392; all *p* < 0.05; Table 6). The intra‐ and interobserver reproducibilities of the ADC histograms were good (0.883–0.919). Therefore, accurately differentiating between active and inactive TAO is necessary.

**Table 6:** ADC histogram and CAS correlation.

	*r*	*p*
Median (μm^2^/s)	0.390	< 0.001
Minimum (μm^2^/s)	0.305	< 0.001
Maximum (μm^2^/s)	0.161	< 0.001
Mean (μm^2^/s)	0.337	< 0.001
Standard deviation	0.027	0.476
Skewness	−0.214	< 0.001
Kurtosis	0.012	0.758
Entropy	0.043	0.258
Percentile		
1st (μm^2^/s)	0.048	0.207
5th (μm^2^/s)	0.350	< 0.001
10th (μm^2^/s)	0.382	< 0.001
25th (μm^2^/s)	0.391	< 0.001
75th (μm^2^/s)	0.389	< 0.001
90th (μm^2^/s)	0.364	< 0.001
95th (μm^2^/s)	0.268	< 0.001
99th (μm^2^/s)	0.211	< 0.001
Median (μm^2^/s)	0.141	< 0.001

*Note:* 1st, 5th, 10th, 25th, 75th, 90th, 95th and 99th: 1st, 5th, 10th, 25th, 75th, 90th, 95th, and 99th percentiles of ADC histograms.

Abbreviations: ADC, apparent diffusion coefficient; CAS, clinical activity score.

## 4 Discussion

Recently, various MRI examinations have been utilized for the diagnosis of TAO with good results, particularly in patients with mild inflammation or inactive stages, which are difficult to observe in clinical settings [13, 14, 15, 16]. Using the TSE‐DWI sequence in orbital imaging studies of patients with TAO, we found that TSE‐DWI can quantitatively assess inflammation in the EOMs. ADC histograms demonstrated good diagnostic efficacy in distinguishing activity TAO, with the 10th percentile improving clinical diagnostic performance. Independent predictors of active TAO included the median, entropy, and SD of the ADC histogram, as well as smoking history and male sex.

The pathophysiological processes of hyaluronic acid accumulation, adipose tissue expansion, and fibrosis occur throughout TAO development [17]. During the active phase of TAO, hydrophilic hyaluronic acid penetrates the interstitial space of the EOMs, leading to edema. During the inactive phase, the EOMs mainly exhibit adipose tissue expansion, collagen deposition, and fibrosis [17, 18]. Increased water in the EOMs during the active phase contributed to elevated ADC [19], whereas fibrosis and collagen deposition in the EOMs during the inactive phase led to decreased ADC. In our study, we found that the ADC distribution of EOMs was clustered in small percentiles: in the 1st to 99th percentiles, the median, mean, minimum, and maximum values in the ADC histograms were significantly higher in the active than that in the inactive TAO group. Previously, elevated ADC in the EOMs of patients with TAO was similarly found using conventional echo planar (EP)‐DWI imaging [10]. In the study, due to artifacts and distortions in the EP‐DWI images, the accuracy of outlining the regions of interest may be reduced, thus affecting the ADC measurement. In our study, compared with the EP sequence, although the signal‐noise ratio of TSE‐DWI was reduced and the scanning time was prolonged, the DWI image artifacts and distortions were reduced, which provided us with better, reproducible, and reliable diffusion‐weighted images and quantitative parameters [20].

Noted by endocrinologists and ophthalmologists, TAO is a common clinical problem, and assessment of inflammation is essential for the treatment and management of patients with TAO [5, 21]. Prompt treatment is required if TAO causes marked ocular discomfort and/or changes in appearance, and hormonal shock therapy is a better option when TAO is active [22]. MR performs an important role in assessing TAO activity. For example, T2WI signal intensity ratio (SIR) [23] and Dixon [24] can evaluate TAO activity.

However, SIR results may vary depending on the reference tissue used for comparison (e.g., temporal muscle, white matter, and thalamus) [25], whereas Dixon has a 4%–14% fat–water exchange failure rate and is motion‐sensitive [24, 26]. These limitations reduce their utility in TAO and compromise staging accuracy. Routine ADCs offer valuable insights for diagnosing and staging TAO [27]. However, the duration, severity, and pathophysiological involvement of each EOM in TAO patients are inconsistent. Conventional ADC values provide only averages and fail to reflect the internal heterogeneity of the EOMs [9, 10]. Therefore, assessment of TAO activity or inactivity using mean values may need improvement. Histograms can provide a more detailed analysis of TAO activity by rationally selecting optimization parameters based on the distribution of the ADC.

Therefore, to provide more clinical information into EOMs and to differentiating TAO activity, we analyzed the ROC curves of ADC histograms to distinguish active from inactive TAO. Our results demonstrated that the ADC histogram effectively differentiated TAO activity. Smaller percentiles showed better diagnostic performance than larger percentiles, with the median outperforming the mean. Notably, the 10th percentile and median exhibited the highest diagnostic efficacy, with the 10th percentile significantly enhancing the diagnostic accuracy of clinical factors (age, sex, and smoking in the joint model). These findings are crucial for helping clinicians accurately differentiate between active and inactive TAOs, enabling appropriate interventions and treatments.

In our study, we identified male sex and smoking as risk factors for active TAO. Oeverhaus et al. [28] reported that male TAO patients who smoked experienced more severe disease. Similarly, Yang and He [29] observed that EOMs are more affected in male and older TAO patients, with a higher prevalence of involvement during the active phase. Smoking is a key factor driving TAO progression and exacerbation [22]. In smokers, the overexpression of IL‐6, IL‐1B, and IEGs activates pathways linked to adipogenesis and inflammatory responses, accelerating the progression of TAO [30]. Therefore, smoking cessation is strongly recommended for all TAO patients to mitigate the risk of TAO progression [22].

In this study, we also found that a median > 1.26 μm^2^/s, entropy > 4.03, and SD > 0.4 were independent predictors of active TAO. A higher median ADC indicated more severe inflammatory edema in EOMs. SD reflected the degree of dispersion of the data, with a higher SD indicating a more heterogeneous distribution of ADC in EOMs. Entropy reflects the randomness and average information of the histogram [31], and higher entropy similarly indicates a more heterogeneous distribution of ADCs in the EOMs. Therefore, the possibility of active TAO must be considered when the degree of inflammatory edema in EOMs is high and the uneven distribution of ADC is obvious.

In the present study, we assessed the correlation between the CAS and ADC histograms in patients with TAO. We found that the CAS did not correlate, or poorly correlated, with the ADC histogram values of EOMs. Previous studies have similarly found no significant correlation between the ADC and CAS [10, 15]. The CAS is more responsive to the overall appearance and symptoms of the patient's eye, whereas the ADC histogram is based on DWI imaging, which responds only to edema of the EOMs. However, some researchers have suggested a significant correlation between ADC and CAS [13]. This discrepancy may be related to the fact that their study [13] included only EOMs with the strongest signals, whereas we included all EOMs in our study.

This study has some limitations. First, despite implementing necessary measures, selection bias inherent to retrospective studies remains unavoidable, highlighting the need for large‐sample prospective studies for further validation. Second, as a single‐center study with a relatively small sample size, additional data from larger, multicenter studies are required to strengthen the findings. Finally, the study did not include follow‐up data on the treatment outcomes or prognosis of patients with TAO.

In summary, a 10th percentile of the TSE‐DWI‐based ADC histogram > 0.78 was effective in differentiating active from inactive TAO and improved diagnostic efficacy for age, male sex, and uptake. The likelihood of having active TAO was high if male sex and smoking and if the ADC of EOMs had a median > 1.26 μm^2^/s, an entropy > 4.03, or an SD > 0.4. ADC histograms may be a useful functional MRI technique for assisting clinicians in understanding the orbital onset of TAO.

## Author Contributions

**Defu Li:** writing–original draft, conceptualization, data curation, funding acquisition, methodology, project administration, resources. **Tingting Zhu:** writing–original draft, conceptualization, formal analysis, investigation, supervision. **Yujin Wang:** writing–original draft, software, validation, visualization. All authors reviewed the text and approved the final version of the manuscript for publication.

## Ethics Statement

The Ethics Committee of Fuyong People's Hospital of Baoan District, Shenzhen, China, approved this study.

## Consent

The need for informed consent was waived owing to the retrospective nature of the study.

## Conflicts of Interest

The authors declare no conflicts of interest.

## Data Availability

The data sets generated during and/or analyzed during this study are not publicly available but are available from the corresponding author upon reasonable request.
